# Alphacoronaviruses Detected in French Bats Are Phylogeographically Linked to Coronaviruses of European Bats

**DOI:** 10.3390/v7122937

**Published:** 2015-12-02

**Authors:** Anne Goffard, Christine Demanche, Laurent Arthur, Claire Pinçon, Johan Michaux, Jean Dubuisson

**Affiliations:** 1 Molecular & Cellular Virology, University Lille, CNRS, Inserm, CHU Lille, Institut Pasteur de Lille, U1019-UMR 8204-CIIL-Centre d’Infection et d’Immunité de Lille, Bâtiment IBL. 1 rue du Pr. Calmette CS 50447, 59021 Lille Cedex, France; jean.dubuisson@ibl.cnrs.fr; 2 Bacterial Respiratory Infections: Pertussis and Tuberculosis, University Lille, CNRS, Inserm, CHU Lille, Institut Pasteur de Lille, U1019-UMR 8204-CIIL-Centre d’Infection et d’Immunité de Lille, F-59000 Lille, France; christine.demanche@univ-lille2.fr; 3 Museum d’Histoire Naturelle de Bourges, Les Rives d’Auron, allée René Ménard, 18000 Bourges, France; laurent.arthur@ville-bourges.fr; 4 University Lille, CHU Lille, EA 2694-Santé publique: épidémiologie et qualité des soins, F-59000 Lille, France; claire.pincon@univ-lille2.fr; 5 Conservation Genetics Unit, Institute of Botany (B. 22), University Liège, 4000 Liège, Belgium; johan.michaux@ulg.ac.be; 6 CIRAD TA C-22/E-Campus international de Baillarguet, 34398 Montpellier Cedex 5, France

**Keywords:** bats, alphacoronavirus, coronavirus, phylogeographic analysis, phylogenetic analysis, Europe, molecular characterization

## Abstract

Bats are a reservoir for a diverse range of viruses, including coronaviruses (CoVs). To determine the presence of CoVs in French bats, fecal samples were collected between July and August of 2014 from four bat species in seven different locations around the city of Bourges in France. We present for the first time the presence of alpha-CoVs in French *Pipistrellus pipistrellus* bat species with an estimated prevalence of 4.2%. Based on the analysis of a fragment of the *RNA-dependent RNA polymerase* (*RdRp*) gene, phylogenetic analyses show that alpha-CoVs sequences detected in French bats are closely related to other European bat alpha-CoVs. Phylogeographic analyses of *RdRp* sequences show that several CoVs strains circulate in European bats: (i) old strains detected that have probably diverged a long time ago and are detected in different bat subspecies; (ii) strains detected in *Myotis* and *Pipistrellus* bat species that have more recently diverged. Our findings support previous observations describing the complexity of the detected CoVs in bats worldwide.

## 1. Introduction

In 2012, a novel coronavirus (CoV), the Middle East Respiratory Syndrome (MERS)-CoV, emerged in humans in the Arabian Peninsula [1]. This CoV is highly pathogenic, as was the Severe Acute Respiratory Syndrome (SARS)-CoV that has emerged in 2002/2003 in China, causing a worldwide outbreak with 774 deaths [2]. CoV belongs to the subfamily of *Coronaviridae* in the order of *Nidovirales*. CoVs are divided into four genetic and serologic genera: alpha- and beta-CoVs, that infect mammals, and gamma- and delta-CoVs known to infect mainly birds [3].

Bats, in the order of *Chiroptera*, are widely distributed across various ecosystems. They constitute one of the largest groups of mammals, second in number of species after *Rodentia* and first in terms of individuals present on Earth [4]. Bats are the only mammals that can fly. They fly to hunt, to change their habitat for hibernation and to migrate. However, less than 3% of extant bat species show migratory movements greater than 50 km [5]. The order of *Chiroptera* is divided into two suborders: *Megachiroptera* and *Microchiroptera* [6]. *Microchiroptera* were reported to live in Europe with 53 species described [6]. European bats inhabit temperate regions and use torpor and hibernation during winter. Chiropters occupy diversified habitats from cities to the countryside and exhibit a large diversity of diets. Their different diets led bats to colonize various ecosystems. In Europe, *Microchiroptera* are mainly considered as insectivorous. They nest in attics, barns, or unoccupied buildings but also in rocks, trees, barks, hollows, and under leaves [7]. In Europe, bats are often the only wild mammals living in human habitats. Some bat species are solitary but, frequently, they form colonies that can reach a million of individuals. In France, 34 bat species have been described and two species, *Pipistrellus pipistrellus* and *Eptesicus serotinus*, are present in the whole territory. Bats have been considered as the natural hosts of many common animal and human viruses, such as measles or mumps, and now they have been considered to be natural reservoirs for SARS-CoV and MERS-CoV (reviewed in [8]).

Phylogeography has been defined as the “field of study concerned with the principles and processes governing the geographical distributions of geographical lineages, especially those within and among closely related species” [9]. The tools of phylogeography have been applied to various fields of biology, such as biodiversity, and recently to explore the links between human migration and viral outbreaks [10]. Thus, using phylogeographic analyses, it has been shown that the HIV outbreak was due to repeated introductions of simian immunodeficiency viruses (SIVs) in the human population [11]. These tools are also used to study the spread of the flu outbreak in 2009 or to speculate on the origin of human hepatitis B [12,13]. Therefore, phylogeography proposes a set of tools that can help to understand how CoV circulate among an animal population, such as uropean bats as described here.

Since 2008, alpha- and beta-CoVs have been identified in several European countries from various bat species, however, no data are available on the presence of CoVs in French bats, as proved by the consultation of the database of bat-associated viruses (http://www.mgc.ac.cn/DBatVir/) [14,15,16,17,18,19]. The aim of this study is to describe alpha-CoVs among French bats, especially in *Pipistrellus pipistrellus*, one of the most common bats in France. To our knowledge, we present the first report of alpha-CoV RNA detection in French bats and the phylogeographic relationships between European alpha-CoVs, based on the analyses of previously published sequences of bat alpha-CoVs.

## 2. Materials and Methods

### 2.1. Study Area and Sampling

**Figure 1:**
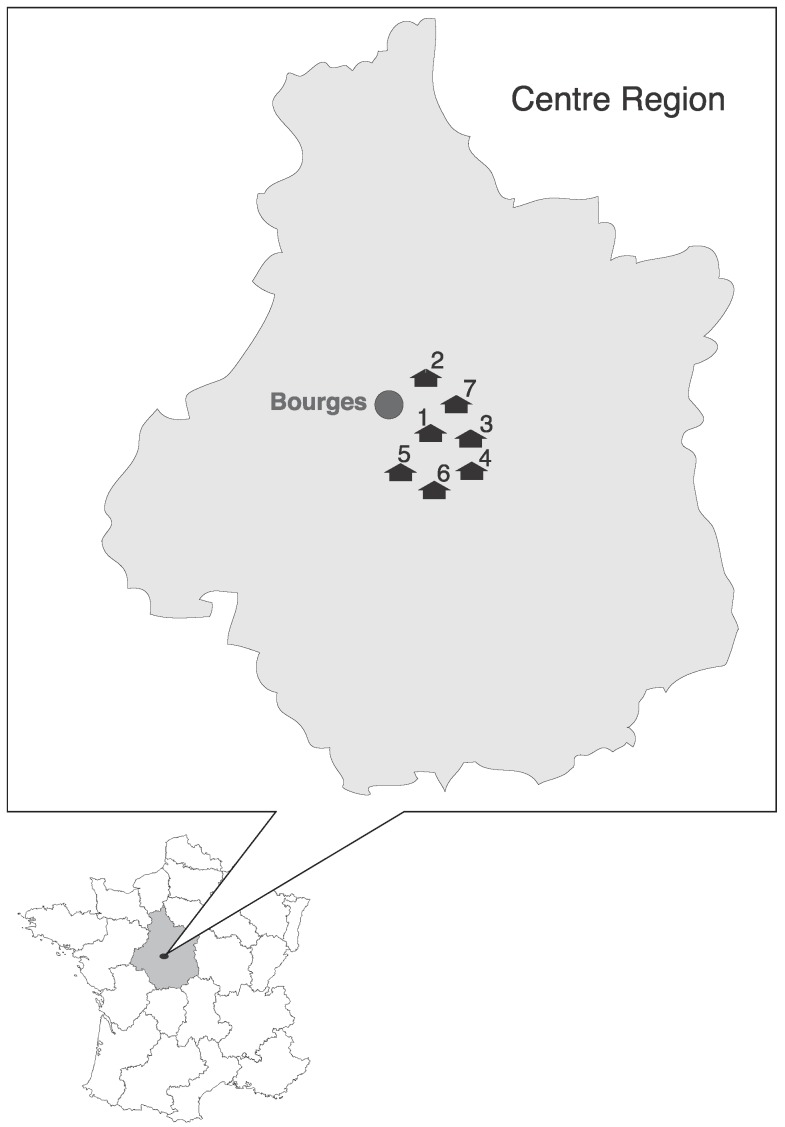
Geographical locations of bat colonies where guano samples were taken during the summer of 2014. The colonies are numbered 1 to 7 and georeferenced as: 1 (02°34′54″ E; 47°00′39″ N), 2 (02°31′02″ E; 47°04′59″ N), 3 (02°32′19″ E; 46°56′23″ N), 4 (02°33′38″ E; 47°02′39″ N), 5 (02°31′22″ E; 47°04′24″ N), 6 (02°18′59″ E; 46°51′27″ N), and 7 (02°23′21″ E, 46°49′28″ N). They are scattered around the city of Bourges, in the central region of France.

Samples were collected from seven distinct locations, each harboring a single bat species, during July and August of 2014. Colonies were located near Bourges in the central region of France (Figure 1). The roosts of bat colonies were mainly human dwellings, such as attics and barns (Table 1). Around 10 to 40 individuals constituted each colony. During the period of collection, males, pregnant and lactating females, as well as young animals born that year inhabited in the same bat colony. The bats species were identified based on their morphologic characteristics according to the European bat identification keys [20].

A total of 162 guano samples were collected from four bat species, *Pipistrellus pipistrellus* (118 specimens), *Barbastella barbastellus* (24 specimens), *Myotis myotis* (10 specimens) and *Eptesicus serotinus* (10 specimens) (Table 1), as previously described [21,22]. To collect bat guano samples, clean plastic sheets were laid down on flat surfaces beneath bat roosts before sunset. Three days later, fresh guano samples were collected and preserved in 250 µL of RNAlater (Applied Biosystems, Courtabœuf, France) during shipment by mail. Several fecal samples were harvested for each colony. On receipt, samples were stored at −80 °C until analysis.

**Table 1:** Prevalence of alpha-CoV in French bat species.

			Positive Samples/Total of Tested Samples by Location							Total of Positive Samples/Total of Tested Samples	% of Positive	Sequences	Coronavirus Group
Bat Species	Size of Bat Colonies *	Day Roost	1	2	3	4	5	6	7	All Areas			
*Pipistrellus pipistrellus*	Small and Medium	Attics, Barns	3/53	2/20	0/10	0/35				5/118	4.2	Ppip1_FR_2014, Ppip2_FR_2014, Ppip3_FR_2014	α
*Barbastella barbastellus*	Small	Attic							0/24		0		
*Myotis myotis*	Small	Barn					0/10				0		
*Eptesicus serotinus*	Small	Attic						0/10			0		
Total										5/162	3.1		

* Bat colony size was scored as follows: small, 10 to 30 individuals; medium, 31 to 200 individuals; large, >200 individuals.

### 2.2. Genome Detection and Sequencing

Fecal pellets stored in RNAlater were tested after mechanical lysis using a MagNAlyser (Roche Diagnostics, Meylan, France) according to the manufacturer’s instructions. Viral RNA was extracted from 100 µL of fecal homogenate using a viral RNA mini kit and eluted in 50 µL of elution buffer (Qiagen, Courtabœuf, France). Samples were then analyzed for the presence of CoV RNA using a nested reverse transcription (RT)-PCR targeting the RNA-dependent RNA polymerase (*RdRp*), slightly modified from Souza *et al*. [23]. RNA (5 µL) was random primed reverse transcribed (High Capacity cDNA Reverse Transcription kit; Applied Biosystems). Twenty-five microliters of reactions were carried out using Taq DNA polymerase (New England Biolabs, Evry, France) with 2 μM of sense and anti-sense primer, and 5 μL of complementary DNA (cDNA). Thermal cycling was set at 94 °C for 1 min and then 40 cycles of 94 °C for 30 s, 50 °C for 30 s, 68 °C for 40 s, and final extension at 68 °C for 5 min. The nested PCR protocol, unmodified from Souza *et al*. [23], used 1 μL of first round PCR product. Negative and positive controls were included in each experiment, in DNA extraction, reverse transcription, DNA PCR, and nested PCR amplifications. Amplicons were purified using the NucleoSpin Gel and PCR clean-up kit (Macherey-Nagel, Hoerdt, France). Purified products were cloned using TOPO TA cloning kit for subcloning with TOP10F’ *E. coli* (Life Technologies, Illkirch, France). Three positive clones of each amplicon were sent for sequencing using M13 forward and reverse primers to Genoscreen (Pasteur Campus, Genopole of Lille, Lille, France).

### 2.3. Sequence Analysis

The *RdRp* gene sequences described in this study were initially aligned with homologous sequences of alpha-CoVs from humans, civet, camel, and bats (Table 2) using CLUSTAL X v1.63b [24]. The aligned sequences were converted to distance matrix (% of differences) using PAUP 4.0b10 software [25]. Maximum likelihood (ML) analyses of sequences were carried out with PhyML v3.0 [26] using the GTR (general time reversible) + Γ (gamma distribution of rates with four rate categories) + I (proportion of invariant sites) model. The appropriate model of sequence evolution was selected using PhyML with automatic model selection by Smart Model Selection (SMS) to determine the evolutionary model which best fits the input data [27]. Evaluation of statistical confidence in nodes was based on 1000 bootstrap replicates [28]. Alignments of polymerase gene sequences used in the various analyses are available upon request from the corresponding author.

### 2.4. Phylogeographic Analysis

A minimum spanning network was constructed using the MINSPNET algorithm available in the ARLEQUIN 2.0 program [29]. The genetic divergences between sample groups were estimated using a distance analysis (K_2_P, mega program).

**Table 2:** List of sequences used for phylogeny analyses with Genbank accession number, coronavirus group, host species and geographic origin and name used in this study.

GenBank Accession Number	Group	Host Species	Geographic Origin	Name	Reference
AY903459	β	Human	Belgium	OC43_BEL_2003	[30]
KC243392	β	*Pipistrellus nathusii*	Ukraine	Pnat_UKR_2011	[31]
KC243391	β	*Pipistrellus nathusii*	Romania	Pnat_ROM_2009	[31]
KF906251	β	Dromedary	United Arab Emirates	Dro_UAE_2013	[14]
JX869059	β	Human	Saudi Arabia	MERS_SAU_2012	[32]
EF507780	β	Human	France	HKU1_FR_2005	[33]
GU190221	β	*Rhinolophus euryale*	Bulgaria	Reur2_BLG_2008	[17]
FJ588686	β	*Rhinolophus sinicus*	China	Rsin_CHI_2006	[34]
AY304488	β	Civet	China	Civ_CHI_2003	[35]
KJ652334	α	*Myotis daubentonii*	Hungary	Mdau_HUN_2013	[19]
KJ652333	α	*Myotis nattereri*	Hungary	Mnat_HUN_2013	[19]
KJ652332	α	*Pipistrellus pigmae*	Hungary	Ppig_HUN_2013	[3]
KJ652331	α	*Myotis myotis*	Hungary	Mmyo_HUN_2013	[3]
KJ652330	α	*Rhinolophus ferrumequinum*	Hungary	Rfer_HUN_2013	[3]
KJ652329	α	*Rhinolophus ferrumequinum*	Hungary	Rfer_HUN_2013	[3]
KF500949	α	*Pipistrellus kuhlii*	Italy	Pkuh1_ITA_2010	[18]
KF500945	α	*Pipistrellus kuhlii*	Italy	Pkuh2_ITA_2011	[18]
JF440366	α	*Myotis nattereri*	United Kingdom	Mnat1_UK_2009	[17]
JF440365	α	*Myotis nattereri*	United Kingdom	Mnat2_UK_2009	[17]
JF440353	α	*Myotis daubentonii*	United Kingdom	Mdau1_UK_2009	[12]
JF440351	α	*Myotis daubentonii*	United Kingdom	Mdau2_UK_2009	[12]
JF440349	α	*Myotis daubentonii*	United Kingdom	Mdau3_UK_2009	[17]
HQ184061	α	*Hypsugo savii*	Spain	Hsav_SP_2007	[16]
HQ184060	α	*Pipistrellus* sp.	Spain	Psp_SP_2007	[16]
HQ184058	α	*Pipistrellus kuhlii*	Spain	Pkuh_SP_2007	[16]
HQ184057	α	*Myotis myotis*	Spain	Mmyo_SP_2007	[16]
HQ184056	α	*Myotis daubentonii*	Spain	Mdau_SP_2007	[16]
HQ184051	α	*Nyctalus lasiopterus*	Spain	Nlas_SP_2007	[16]
GU190239	α	*Nyctalus leisleri*	Bulgaria	Nlei_BLG_2008	[36]
GU190237	α	*Rhinolophus euryale*	Bulgaria	Reur1_BLG_2008	[36]
GQ259966	α	*Myotis dasycneme*	The Netherlands	Mdas_NLD_2006	[15]
GQ259964	α	*Pipistrellus pipistrellus*	The Netherlands	Ppip_NLD_2008	[15]
GQ259967	α	*Myotis dasycneme*	The Netherlands	Mdas_NLD_2007	[15]
EU375871	α	*Myotis daubentonii*	Germany	Mdau_GER_2007	[14]
EU375869	α	*Pipistrellus nathusius*	Germany	Pnat1_GER_2007	[14]
EU375868	α	*Pipistrellus pigmae*	Germany	Ppig1_GER-2007	[14]
EU375867	α	*Pipistrellus pigmae*	Germany	Ppig2_GER-2007	[14]
EU375864	α	*Pipistrellus nathusius*	Germany	Pnat2_GER_2007	[14]
EU375863	α	*Myotis dasycneme*	Germany	Mdas_GER_2007	[14]
AY864196	α	*Miniopterus* sp.	China	HKU8_CHI_2004	[37]
DQ249228	α	*Miniopterus* sp.	China	HKU8_CHI_2005	[38]
NC009988	α	*Rhinolophus* sp.	China	HKU2_CHI_2004	[39]

### 2.5. Statistical Analyses

Prevalences of CoV were estimated with 95% confidence intervals constructed using the normal approximation. A Fisher’ exact test was done to compare the prevalence of CoV for *Pipistrellus pipistrellus* to the prevalence for the other species (the other species were considered as a single group since no positive sample had been detected).

### 2.6. Nucleotide Sequence Accession Numbers

*RdRp* gene sequences were deposited in GenBank under accession number KT345294 to KT345296.

## 3. Results

A total of 162 guano samples of bats were collected from seven bat colonies located at seven different sites around the city of Bourges in the central region of France, during the summer of 2014 (Figure 1). Guano collections were all carried out in July, except for colony 7, which was completed in August.

CoV RNA was detected in five out of 162 samples. All the CoV-positive samples were detected from *Pipistrellus pipistrellus*. No CoV RNA was detected from *Barbastella barbastellus*, *Myotis myotis* and *Eptesicus serotinus*. Prevalence of CoV was estimated at 3.1% (CI95%: (0.4%; 5.8%)) in the whole sample, all positive sample being detected in *Pipistrellus pipistrellus*, leading to a prevalence for this species estimated at 4.2% (CI95% : (0.6%; 7.9%)), compared to 0% for the other species; however, this difference in prevalence was not significant (Fisher’ exact test, *p* = 0.32).

**Figure 2:**
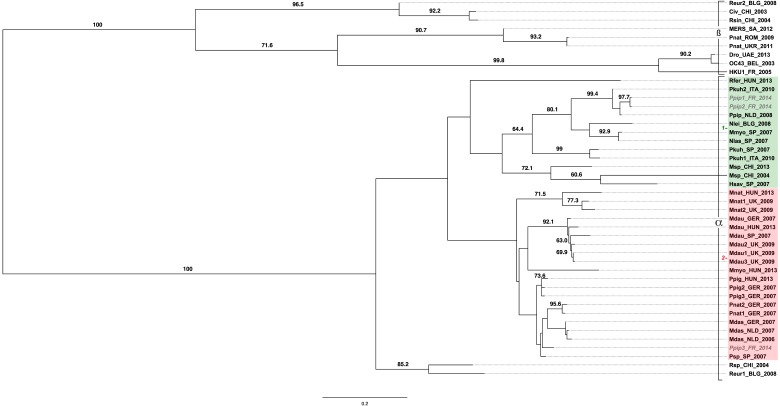
Phylogenetic tree of the partial RNA-dependent RNA polymerase (*RdRp*) gene (277 bp) of coronavirus strains found in bats. The phylogram results from bootstrapped data sets obtained using PhyML 3.0 program [26]. The tree was visualized using the FigTree program, version 1.4.2. The percentages above the branches are the frequencies with which a given branch appeared in 1000 bootstrap replications. Bootstrap values below 60% are not displayed. Taxa are named according to the following pattern: bat species/country of origin/year of detection. Sequences belonging to lineage 1 are presented in the green box, those belonging to lineage 2 in the red box. French bat sequences are presented in grey.

To characterize the overall diversity of CoV sequences, a phylogenetic analysis of bat CoVs was performed using the sequences of a 440 base pairs (bps) PCR amplicon of the *RdRp* gene from three positive samples. Two sequences, Ppip1_FR_2014 and Ppip2_FR_2014, were obtained from guano collected in a same bat colony. The third French sequence, Ppip3_FR_2014, was obtained from guano collected from another bat colony located 12 km from the first site. For the two remaining samples, we were not able to obtain the sequence of the fragment. Nucleotide sequence analysis shows that the bat CoV corresponding to the sequences amplified from French bats belong to alpha-CoV genera (Figure 2). Comparison of the *RdRp*-aligned sequences was carried out on 277 positions, including gaps, for a total of 48 taxa: three original sequences and 45 previously published (Table 2). The beta-CoV and alpha-CoV sequences are clearly separated in two groups supported each by 100% bootstrap value (Figure 2). Our analyses showed that genetic divergence between beta- and alpha-CoV sequences is up to 35%.

Among the alpha-CoV group, sequences appear separated in two lineages: 1 and 2 (Figure 2). Nucleotide divergence between groups 1 and 2 varies from 22.02% to 30% (Figure S1). We also noticed that sequences of alpha-CoV are grouped according to the host species and independently of date or of the sampling location.

Thus, lineage 1 includes several groups of sequences: (i) HKU8 sequences obtained from *Miniopterus* sp. in 2004 and 2013 in China are grouped with the 72.1% bootstrap value; (ii) another group includes sequences obtained from *P. kuhli* in 2007 in Spain, and in 2010 in Italy (99% of bootstrap values); and (iii) both French sequences from *Pipistrellus pipistrellus*, Ppip1_FR_2014 and Ppip2_FR_2014, are closely related to each other (2.90% of nucleotide sequence divergence) and grouped with sequences obtained from *P. pipistrellus* in the Netherlands in 2008 and *P. kuhli* in Italy in 2010 with a 99.4% bootstrap value. Other sequences belong to lineage 1, such as sequences obtained from *R. ferrumequinum* in 2013 in Hungary, from *N. leisleri* in 2008 in Bulgaria, from *M. myotis*, *N. lasiopterus*, and *H. savii* in Spain in 2007.

Lineage 2 includes several groups. The first one includes sequences obtained from *M. nattereri* in 2009 and 2013 in the United Kingdom and Hungary (71.5% bootstrap value); the second one associates sequences obtained from *M. daubentonii* between 2007 and 2013 in several countries (92.1% bootstrap value); the third one corresponds to sequences from *P. pigmae* in 2007 in Germany and in 2013 in Hungary (73.6% bootstrap value); and the fourth one includes sequences from *P. nathusius* in 2007 in Germany (95.6% bootstrap value). Sequences obtained from *M. dasycneme* in 2006 in the Netherlands, and in 2007 in Germany and the Netherlands, are also grouped with low support (bootstrap values smaller than 60%). A French bat sequence obtained from *P. pipistrellus*, Ppip3_FR_2014, also belongs to this last group. Finally, sequences obtained from *M. myotis* in 2013 in Hungary and from *Pipistrellus* sp. in 2007 in Spain are also included in lineage 2.

Finally, two sequences obtained from *Rhinolophus* sp. in 2004 in China and from *R. Euryale* in 2008 in Bulgaria appear highly separated in a divergent lineage, supported by an 85.2% bootstrap value.

**Figure 3:**
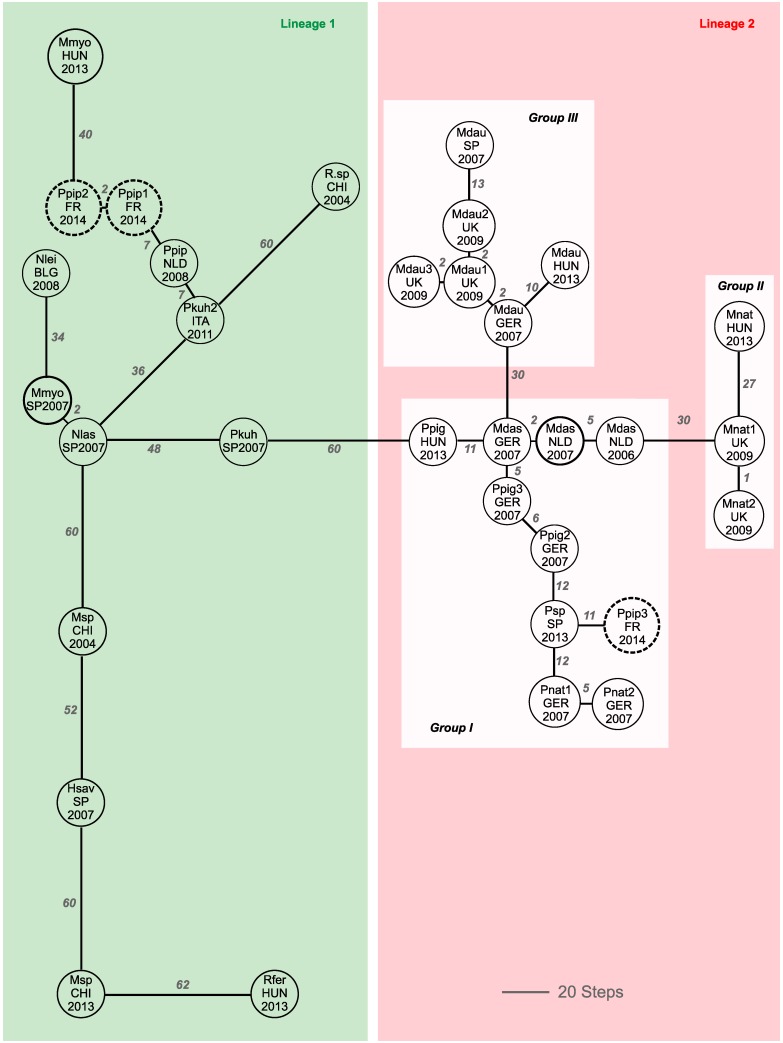
Minimum spanning network constructed using *RdRp* gene sequences of bat alpha-CoV. Bat species and subspecies, geographic origins and year of detection are indicated. Numbers correspond to the mutational steps observed between sequences. Sequences belonging to lineage 1 are presented in the green box, those belonging to lineage 2 in the red box. Among lineage 2, groups I–III are presented in white boxes. French bat sequences are presented in dotted bold circles.

The minimum spanning network illustrates the mutational relationship of the European alpha-CoVs in bats (Figure 3). Thirty-four different alpha-CoV sequences were used for analyses and evidenced several groups. The first group (group I) associates three sequences, closely interconnected with two and five mutational steps obtained from *M. dasycneme* in 2007 in Germany and in 2006 and 2007 in the Netherlands, and seven sequences obtained from several subspecies of *Pipistrellus* in various countries. The French sequence Ppip3_FR_2014, detected from *Pipistrellus* guano, belongs to this group.

Two other groups (groups II and III) are separated from group I with 30 mutational steps each. Group II includes three sequences obtained from *M. natttereri* in 2009 and 2013 in the United Kingdom and Hungary. Group III includes six sequences obtained from *M. daubentonii* in several countries.

The other analyzed sequences appear highly differentiated with important levels of mutational steps among them (from 34 to 62). An exception is nevertheless observed for the sequences Pkuh2_ITA_2010, Ppip_NLD_2008, Ppip1_FR_2014, Ppip2_FR_2014, Nlas_SP_2007, and Mmyo_SP_2007, which appear more closely related with less than eight mutational steps among them.

The topology of the minimal spanning network adopts the same configuration than the phylogenetic tree. Indeed, lineage 2 observed on the tree is characterized by short branches lengths suggesting a recent diversification for these sequences. They also appear closely interconnected in the network, with low levels of mutational steps among them.

In contrast, lineage 1 of the phylogenetic tree is characterized by longer branches lengths, suggesting ancient separations among sequences of this lineage. Important levels of genetic divergence observed between these sequences corroborate this result.

Phylogeographic and phylogenetic analyses therefore give congruent results, although the minimum spanning network sometimes give a better robustness for some groups, represented by lower bootstrap values in the phylogenetic tree.

## 4. Discussion

### 4.1. Prevalence of Alpha-CoV in French *Pipistrellus pipistrellus*

Previous studies using nested RT-PCR reported important differences in prevalence between bat species in several countries. In China, prevalence of CoV RNA in bats varies between 6.5% and 48% [38,39,40]. In Germany, overall prevalence of alpha- and beta-CoVs was reported at 9.8% in different bat species [14]. Concerning the prevalence of alpha-CoV in Europe, it reached 75% in *Myotis nattereri* [14,17]. CoVs are also detected in *Barbastella barbastellus*, in *Myotis myotis*, and *Eptesicus serotinus* species in Europe [17,19,41].

The overall prevalence (3.1% (CI95%: (0.4%; 5.8%)) of alpha-CoV reported here in four bat species, and especially in *Pipistrellus pipistrellus* (4.2% (CI95%: (0.6%; 7.9%)) is lower than in previous observations, but is similar to those reported in *Pipistrellus* sp. in Spain (3.6%) [16]. The differences can be explained by the way the bat guano was collected. Indeed, in previously published studies, animals were caught to obtain biological samples while, in our work, we collected the fresh guano in different bat colonies. We cannot exclude that we may have studied several samples produced by the same individual. Therefore, our results may potentially be explained by the degradation of viral RNA under natural conditions. In our study, no CoVs were detected in *Barbastella barbastellus*, *Myotis myotis* and *Eptesicus serotinus* species. In addition to the potential degradation of viral RNA, our results may be explained by the low number of collected samples, the small number of bat species sampled, regarding the 34 bat species listed in France, and the sampling location being limited to the area near Bourges. To further extend this study, it will be necessary to analyze a larger number of samples, collected in different locations in France, to determine the real prevalence of CoV in French bats.

In humans, alpha-CoV, HCoV-229E, and HCoV-NL63, cause the common cold. Bats are identified as natural reservoir for CoV and, recently, it has been shown that hipposiderid (*Hipposideridae*) bats may be infected with an alpha-CoV closely related to HCoV-229E [42]. However, in our work, phylogenetic analyses with human alpha-CoV sequences, such as HCoV-229E or HCoV-NL63, failed because the genetic divergences were very high (data not shown).

The phylogenetic analyses conducted from a fragment of *RdRp* gene reported here show that alpha-CoV sequences are separated into two major lineages, 1 and 2, and a minor group. The genetic divergence between the two major lineages varies between 20% and 35%. The French sequences are distributed within the two major lineages. Both Ppip1_FR_2014 and Ppip2_FR_2014 sequences detected from guano collected from the same bat colony were closely related, and are included in lineage 1. The third French sequence, Ppip3_FR_2014, obtained from guano collected from another bat colony is included in lineage 2. Both colonies are located by 12 km away from each other. Such result would be explained by the fact that *Pipistrellus* bats are very loyal solitary and that they usually remain confined to their own colonies, even if another colony is located 10 km away. Similar observations have been reported for *P.* *nathusius* in Germany or *M. nattereri* in the United Kingdom [14,17]. Thus, the phylogenetic tree presented here shows several clusters of sequences grouped by bat species. These results confirm the existence of coronavirus strains specific to bat species and suggest a low circulation of viral strains among bat species [14,15,17].

On the basis of genetic diversity, seven lineages of alpha-CoV have been previously described in Europe [14,15,17]. The previously described lineages, 1–4, are distributed among lineage 2, which we have defined. The genetic diversity among alpha-CoV sequences obtained from bats was very high (up to 45.5%). Our results show that sequences obtained from several bat species in different European countries are grouped together. A new definition of bat coronavirus lineage seems to be required to describe the diversity of alpha-CoV in Europe.

### 4.2. Phylogeographic Relatedness among Alpha-CoVs Detected in European Bats

Phylogeography is used to study the circulation of an infectious agent and an animal population or the dissemination of an infectious agent in a group of humans [43,44]. Here we used this tool to describe the circulation of alpha-CoV among European bats. The topology of the minimum spanning network shows that two types of alpha-CoVs strains circulate in European bats. On the one hand, the old strains diverged a long time ago from a common unknown ancestor, as suggested by the large mutational steps observed between sequences belonging to lineage 1. The identification of the common ancestor of alpha-CoVs strains may be difficult since the diversification of European bats and perhaps bat viruses date to the glacial periods [45]. On the other hand, the strains detected in *Myotis* and *Pipistrellus* bat species, which are interconnected with smaller mutational steps, have more recently diverged. These strains may have been recently introduced in the European bat populations and have quickly circulated within *Myotis* and *Pipistrellus* bat species. This recent introduction may explain why these strains are specific to their host as suggested by previous studies [15,16].

In conclusion, previous studies showed the presence of alpha-CoV in various European bat species. However, to our knowledge, this is the first report describing the presence of alpha-CoV RNA in French bat species, and the first description of phylogeographic relatedness among alpha-CoV detected in European bats. Our findings support previous observations describing the complexity of the detected CoVs in bats in Europe, but also in South America, China, and Eastern Thailand [40,46,47].

## Supplementary Files

Supplementary File 1
