# Supplementary material for: Alphacoronaviruses Detected in French Bats Are Phylogeographically Linked to Coronaviruses of European Bats
Source: Viruses. 2015 Dec 2;7(12):6279–90. doi: 10.3390/v7122937 (PMC4690861; doi:10.3390/v7122937)
Supplement: Supplementary File 1 [file viruses-07-02937-s001.doc]

**Supplementary information**

1 2 3 4 5 6 7 8 9 10 11 12 13 14 15 16 17 18 19 20 21 22 23 24 25 26 27 28 29 30 31 32 33 34 35 36 37 38 39 40 41 42 43

1 Pnat_UKR_2011

2 Pnat_ROM_2009 00.36

3 MERS_UAE_2012 11.19 11.19

4 Rsin_CHI_2006 37.55 37.55 37.55

5 Civ_CHI_2003 38.99 38.99 37.91 03.61

6 Reur2_BLG_2008 34.66 34.30 34.66 13.00 12.64

7 Reur1_BLG_2008 44.30 40.70 41.16 44.30 38.63 37.55

8 HKU2_CHI_2004 41.16 47.90 39.71 38.27 37.18 38.63 14.44

**9 Ppip2_FR_2014** 47.9 44.30 43.68 47.90 47.90 40.43 29.60 27.08

**10 Ppip1_FR_2014** 47.90 44.30 43.68 47.90 47.90 40.43 29.60 27.08 00.72

11 Ppip_NLD_2008 40.70 39.71 42.96 42.24 42.24 40.79 29.24 26.71 02.89 02.89

12 Pkuh2_ITA_2010 38.99 38.63 42.24 42.24 42.24 40.79 29.24 26.71 03.97 03.97 02.17

13 Mmyo_SP_2007 36.82 36.46 39.35 42.96 42.60 40.79 26.35 24.55 15.88 15.88 14.44 13.72

14 Nlas_SP_2007 36.82 36.46 40.7 42.96 42.60 40.79 26.35 24.55 15.88 15.88 14.44 13.00 00.72

15 Nlei_BLG_2008 37.91 37.55 47.90 45.13 44.04 42.96 23.83 23.10 16.25 16.25 15.16 15.88 11.91 12.64

16 Pkuh_SP_2007 38.27 37.91 39.71 38.27 37.55 36.10 25.99 23.10 19.13 19.13 19.13 19.13 16.97 16.97 20.22

17 Pkuh1_ITA_2010 38.63 38.27 44.3à 38.63 37.91 38.27 25.63 22.38 18.05 18.05 19.49 19.49 18.41 18.41 18.77 03.97

18 Hsav_SP_2007 38.99 38.63 38.99 43.68 43.68 40.79 26.35 27.44 25.27 24.55 24.55 25.27 25.63 25.63 24.55 22.38 24.91

19 HKU8_CHI_2004 40.70 40.70 38.63 42.60 40.79 40.79 25.63 27.44 24.91 24.19 24.91 24.19 24.19 24.91 23.47 27.08 26.71 21.66

20 HKU8_CHI_2005 37.18 36.82 38.27 42.60 41.88 38.27 23.83 24.91 21.30 21.30 20.94 20.94 20.58 20.58 23.47 20.58 20.58 18.77 2310

21 Rfer_HUN_2013 40.07 40.07 40.07 40.79 40.79 41.88 26.35 29.60 26.71 26.71 26.35 27.08 24.91 24.91 28.16 25.27 26.35 24.91 22.74 27.08

22 Ppig1_GER_2007 41.52 41.52 38.63 38.99 38.63 38.99 25.99 24.91 28.16 28.16 26.71 26.71 24.55 24.55 27.08 22.38 2.310 27.44 28.88 24.55 24.55

23 Ppig2_GER_2007 41.88 41.88 38.99 39.71 39.35 38.99 27.08 24.55 27.44 27.44 25.99 25.99 24.19 24.19 27.08 22.38 23.10 28.16 28.88 25.27 25.27 01.44

24 Ppig_HUN_2013 41.88 41.88 38.63 39.35 38.99 38.63 27.08 24.55 27.08 27.08 25.63 25.63 23.83 23.83 25.99 20.94 21.66 28.16 28.16 24.91 24.91 02.17 02.17

**25 Ppip3_FR_2014** 41.52 41.52 37.18 38.27 37.55 37.91 27.44 25.63 28.88 28.88 27.08 27.08 24.55 24.55 27.08 25.27 25.99 28.88 28.52 25.63 26.35 05.42 05.42 06.14

26 Psp_SP_2007 40.79 40.79 37.18 38.27 37.91 38.27 25.99 23.83 27.80 27.80 26.35 26.35 24.19 24.19 26.35 23.47 23.47 29.96 29.60 24.19 24.91 03.97 03.97 04.69 03.97

27 Pnat2_GER_2007 40.07 40.07 37.55 40.07 40.43 40.43 25.27 25.27 28.52 28.52 27.08 26.35 23.47 23.47 25.99 22.38 23.83 28.88 29.24 24.55 24.55 06.86 06.86 06.86 07.22 06.14

28 Pnat1_GER_2007 39.71 39.71 37.18 40.07 40.43 40.07 26.71 25.27 28.16 28.16 25.99 25.99 24.55 24.55 27.08 24.19 25.63 28.88 30.69 24.19 25.99 07.22 07.22 07.22 06.86 05.78 01.81

29 Mdas2_NLD_2007 38.99 38.99 36.82 37.55 38.27 38.63 25.99 25.63 27.44 27.44 25.99 26.71 24.91 24.91 28.88 24.91 24.91 27.80 27.80 23.10 24.19 07.22 07.94 08.30 07.94 07.58 07.94 07.58

30 Mdas1_NLD_2007 38.99 38.99 37.18 37.91 38.63 38.27 25.27 25.27 27.80 27.80 26.35 26.35 23.83 23.83 28.52 24.55 24.55 28.16 27.44 24.19 23.83 06.86 07.58 07.94 07.58 07.22 07.58 07.94 01.44

31 Mdas_GER_2007 39.35 39.35 36.46 37.55 38.27 38.99 25.63 25.27 28.52 28.52 27.08 27.08 24.55 24.55 28.52 24.55 24.55 27.80 27.80 24.19 23.83 06.14 06.86 07.22 06.86 06.50 06.86 07.22 01.44 00.72

32 Mdau3_UK_2009 41.52 41.52 38.63 40.07 38.99 41.88 27.44 25.27 26.35 26.35 24.19 24.19 23.47 23.47 27.44 23.47 23.83 27.08 28.52 23.10 27.44 13.72 13.72 13.36 13.72 14.08 13.72 13.36 11.55 13.00 12.27

33 Mdau1_UK_2009 41.52 41.52 38.63 40.07 38.99 41.88 27.08 24.91 25.99 25.99 23.83 23.83 23.83 23.83 27.08 23.10 23.47 26.71 28.52 22.74 27.44 13.72 13.72 13.36 13.72 14.08 13.72 13.36 11.55 13.00 12.27 00.36

34 Mdau2_UK_2009 41.52 41.52 38.63 39.35 38.27 41.52 26.71 24.55 24.91 24.91 24.19 24.19 24.91 24.91 26.71 22.02 22.38 26.35 27.80 22.74 26.71 14.08 14.08 13.72 14.44 14.44 14.08 14.44 11.91 13.36 12.64 01.44 01.08

35 Mdau_HUN_2013 41.16 40.79 38.99 40.79 40.43 41.88 28.52 25.63 26.35 26.35 24.91 24.91 24.55 24.55 28.88 23.47 23.83 27.44 29.60 23.83 28.88 15.16 15.16 14.80 15.52 15.52 15.16 14.80 13.00 14.44 13.72 03.25 03.25 03.61

36 Mdas_GER_2007 41.16 41.16 38.27 39.35 38.99 40.79 28.16 23.83 23.83 23.83 22.38 22.38 23.10 23.10 27.08 21.66 22.02 26.35 27.80 23.47 26.71 13.72 13.72 12.64 14.08 14.08 13.72 12.64 11.55 13.00 12.27 02.53 02.17 02.53 03.25

37 Mdau_SP_2007 41.52 41.52 38.63 40.07 40.07 42.60 28.52 26.35 26.35 26.35 25.27 26.71 26.71 26.71 29.96 23.83 2419 25.99 28.52 23.47 27.08 14.44 14.44 14.08 14.44 15.16 15.52 15.88 12.64 13.36 12.64 05.42 05.05 04.69 06.86 05.78

38 Mnat2_UK_2009 40.07 40.43 38.99 39.71 40.07 39.35 27.80 27.44 27.08 27.08 26.35 25.63 25.27 25.27 29.60 24.91 25.63 27.08 28.88 23.47 25.63 15.16 15.88 15.88 16.61 15.88 16.97 17.33 14.08 13.72 14.44 16.97 16.61 16.97 18.77 17.33 17.69

39 Mnat1_UK_2009 39.71 40.07 39.35 38.99 39.35 39.71 28.52 28.52 27.44 27.44 26.71 25.99 25.99 25.99 29.24 25.27 25.27 27.80 29.24 22.02 26.35 14.44 15.16 15.16 15.88 15.16 16.25 17.33 13.36 13.00 13.72 16.97 16.61 16.25 18.77 17.33 16.25 04.33

40 Mnat_HUN_2013 37.91 38.27 38.99 37.18 37.91 36.82 28.88 25.99 28.16 28.16 26.35 26.35 24.55 25.27 28.88 25.27 25.99 28.88 28.16 22.38 28.16 16.97 17.33 17.33 18.05 16.25 15.16 15.52 14.44 1408 14.80 17.33 17.33 17.69 19.49 18.05 18.77 11.91 10.47

41 Mmyo_HUN82013 37.55 37.18 36.82 37.55 38.99 37.55 25.27 25.99 26.71 25.99 26.35 25.63 23.47 24.19 24.91 21.30 23.83 25.63 27.44 23.83 25.99 15.88 15.88 16.61 14.44 16.25 14.80 15.88 15.16 15.52 15.52 16.97 17.33 16.61 17.69 16.97 18.05 19.13 18.41 17.33

42 Dro_UAE_2013 36.82 37.18 36.82 36.82 35.38 37.18 41.52 38.27 42.96 42.96 42.60 42.60 42.24 42.96 44.04 41.52 42.96 41.16 43.68 42.60 44.40 45.13 45.49 45.13 43.68 44.04 43.68 43.68 45.49 44.77 4513 45.49 45.13 44.40 46.21 44.40 44.77 43.32 42.60 42.96 43.68

43 OC43_BEL_2003 36.46 36.82 36.10 36.82 35.38 37.91 41.52 38.99 42.60 42.60 42.24 42.24 42.60 43.32 44.40 40.79 42.24 40.79 43.68 41.88 43.68 44.40 44.77 44.40 43.68 43.68 43.32 43.32 45.13 45.13 44.77 45.13 44.77 44.04 45.85 44.04 44.40 43.32 4260 42.96 43.68 02.53

44 HKU1_FR_2005 34.30 34.66 34.30 37.91 36.46 33.94 39.71 38.99 40.43 39.71 41.52 41.16 38.99 39.71 40.43 38.27 39.71 38.99 40.79 40.43 45.49 44.40 44.40 44.40 44.04 43.32 42.60 43.32 4296 42.24 42.96 46.57 46.21 45.49 47.29 45.49 46.21 4.96 42.24 41.16 42.24 15.88 16.97

**Figure S1.**
